# Reaching Deeper: Absolute In Vivo Thermal Reading of Liver by Combining Superbright Ag_2_S Nanothermometers and In Silico Simulations

**DOI:** 10.1002/advs.202003838

**Published:** 2021-03-03

**Authors:** José Lifante, Yingli Shen, Irene Zabala Gutierrez, Irene Rubia‐Rodríguez, Daniel Ortega, Nuria Fernandez, Sonia Melle, Miriam Granado, Jorge Rubio‐Retama, Daniel Jaque, Erving Ximendes

**Affiliations:** ^1^ Nanomaterials for Bioimaging Group (NanoBIG) Departamento de Fisiología ‐ Facultad de Medicina Universidad Autónoma de Madrid Arzobispo Morcillo 2 Madrid 28029 Spain; ^2^ Nanomaterials for Bioimaging Group (NanoBIG) Instituto Ramón y Cajal de Investigación Sanitaria IRYCIS Ctra. Colmenar km. 9.100 Madrid 28034 Spain; ^3^ Nanomaterials for Bioimaging Group (NanoBIG) Departamento de Física de Materiales ‐ Facultad de Ciencias Universidad Autónoma de Madrid C/Francisco Tomás y Valiente 7 Madrid 28049 Spain; ^4^ Departamento de Química en Ciencias Farmacéuticas ‐ Facultad de Farmacia Universidad Complutense de Madrid Plaza Ramón y Cajal S/N Madrid 28040 Spain; ^5^ IMDEA Nanociencia Ciudad Universitaria de Cantoblanco Faraday 9 Madrid 28049 Spain; ^6^ Department of Condensed Matter Physics Faculty of Sciences University of Cádiz Puerto Real (Cádiz) 11510 Spain; ^7^ Biomedical Research and Innovation Institute of Cádiz (INiBICA) Cádiz 11009 Spain; ^8^ Departamento de Óptica, Facultad de Óptica y Optometría Universidad Complutense de Madrid Arcos de Jalon 118 Madrid 28037 Spain

**Keywords:** deep tissue, liver, luminescence, nanoparticles, nano‐thermometry

## Abstract

Luminescent nano‐thermometry is a fast‐developing technique with great potential for in vivo sensing, diagnosis, and therapy. Unfortunately, it presents serious limitations. The luminescence generated by nanothermometers, from which thermal readout is obtained, is strongly distorted by the attenuation induced by tissues. Such distortions lead to low signal levels and entangle absolute and reliable thermal monitoring of internal organs. Overcoming both limitations requires the use of high‐brightness luminescent nanothermometers and adopting more complex approaches for temperature estimation. In this work, it is demonstrated how superbright Ag_2_S nanothermometers can provide in vivo, reliable, and absolute thermal reading of the liver during laser‐induced hyperthermia. For that, a new procedure is designed in which thermal readout is obtained from the combination of in vivo transient thermometry measurements and in silico simulations. The synergy between in vivo and in silico measurements has made it possible to assess relevant numbers such as the efficiency of hyperthermia processes, the total heat energy deposited in the liver, and the relative contribution of Ag_2_S nanoparticles to liver heating. This work provides a new way for absolute thermal sensing of internal organs with potential application not only to hyperthermia processes but also to advanced diagnosis and therapy.

## Graphical abstract

A new procedure for thermal readout is obtained from the combination of in vivo transient thermometry and in silico simulations. Quantities such as the efficiency of hyperthermia processes and the heat deposited in the liver are determined by a careful analysis. The results have potential applications not only in hyperthermia processes but also in advanced diagnosis and therapy.

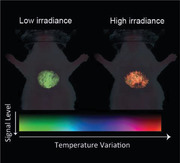

## 1 Introduction

The scientific research in the last decades has attested a widespread ambition on the achievement of minimally invasive in vivo thermal sensing. Contactless thermal sensing could be particularly beneficial for adjusting the delivered energy during the treatment of tumors using photothermal therapy. Such an adjustment, in turn, could result in a reduction of the operative trauma, pain, scarring, recovery time and/or the number of post‐surgical complications.^[^ ^1^ ^]^ The applications of in vivo thermal sensing, however, are not limited to the feedback during the treatment of tumors. Since much is still left to understand about the thermal dynamics of organs during their natural functioning, the urgency of acquiring accurate internal thermal read‐outs seems to be more evident each day. One of the most complex and essential organs in vertebrates is the liver, which has a multifunctional role in maintaining body homeostasis. It performs essential processes including but not limited to complement system metabolism, energy storage in the form of the carbohydrate glycogen, blood detoxification of xenobiotics and endotoxins and nutrient digestion through bile production.^[^ ^2^ ^]^ Thus, the assessment of liver temperature would significantly contributes to the interpretation of its role in body's heat distribution and the effect of temperature in malfunctions such as hepatitis, hemochromatosis, steatosis and cirrhosis.^[^ ^3^, ^4^ ^]^

Despite its critical importance, the contactless evaluation of the thermal dynamics of any internal organ still remains a challenging subject.^[^ ^5^ ^]^ Different strategies are continuously tested including active and passive microwave imaging, ultrasound imaging, magnetic resonance techniques and luminescence thermometry. ^[^ ^6^, ^7^, ^8^ ^]^ Despite the fierce competition, the latter has attracted a great deal of attention in the last years due to the implementation of innovative nanosized luminescent materials, the so‐called luminescent nanothermometers (LNThs).^[^ ^9^ ^]^ LNThs have been successfully employed to access to the thermal dynamics and loadings of cells, tissues and even organs such as brain.^[^ ^10^, ^11^, ^12^, ^13^, ^14^, ^15^ ^]^ One of the most promising advance provided by LNThs was the remote evaluation of the health status of tissues as achieved by the combined use of luminescence‐based transient thermometry (TTh).^[^ ^16^, ^17^, ^18^ ^]^ This technique is based on a simple principle: when a tissue is subjected to a heating cycle, its temperature deviates from its basal (normal) value to a maximum. Once the heating stimulus is off, the tissue starts thermal relaxation until it recovers its basal temperature. The profile of this thermal relaxation obeys to the Penne's bioheat equation and depends on the thermal and physical properties of the tissue.^[^ ^19^, ^20^ ^]^ LNThs make possible non‐invasive measurement of cooling dynamics of the tissue. Any alteration in tissue properties caused by, for example, a pathological context, would alter the cooling profile provided by LNThs.

Although previous works have demonstrated that LNThs and TTh can detect incipient pathologies such as ischemia (in the paw's muscles of mice) and melanoma (superficial), recent results suggest that its applicability cannot be extended to the cases of more internal organs (up to 1–2 cm from the surface in murine models).^[^ ^21^, ^22^ ^]^ The major reason for this limitation is the non‐negligibility of the wavelength‐dependence of tissue‐induced attenuation.^[^ ^23^ ^]^ It has been shown that even in the biological windows (wavelength intervals in the near‐infrared where attenuation of light into tissues is minimized), tissues cause relevant distortions in the emission spectra of LNThs. This, in turn, induces serious errors in the absolute measurement of temperature.^[^ ^24^, ^25^ ^]^ In addition, the temperature dependence of the tissue‐induced attenuation also has a negative impact in the reliability of LNThs when operating at the level of internal organs. The temperature dependent optical properties of tissues lead to an alteration in the thermal sensitivity of LNThs in respect to that determined in an aqueous solution. This not only prevents inferring the absolute value of temperature in a deep organ but also the estimation of temperature variations inside of it.

Thus, given the recently reported drawbacks, two different strategies do exist to determine the liver thermal loading based on LNThs and TTh: i) the use of a thermometric parameter not affected by tissue induced distortions (lifetime, for instance) or ii) to explore alternative methods for the proper interpretation of the cooling dynamics provided by LNTs without requiring the use of re‐calibration. In this work, we evaluate the second approach. We have combined in vivo experimental cooling profiles of mice liver with in silico simulations. Combination of in vivo and in silico data makes possible to determine the absolute liver temperature during a photothermal process. In vivo measurements were performed by using the state of the art in LNThs: we have used Ag_2_S superdots produced by ultrafast photochemistry.^[^ ^26^ ^]^ Although the potential of Ag_2_S superdots for low dose in vivo imaging has been already demonstrated, this work constitutes their debut as thermal transducers and sensors at the in vivo level.

## 2 Results and Discussion

### 2.1 Characterization of Ag_2_S Superdots as TTh Reporters

**Figure 1:**
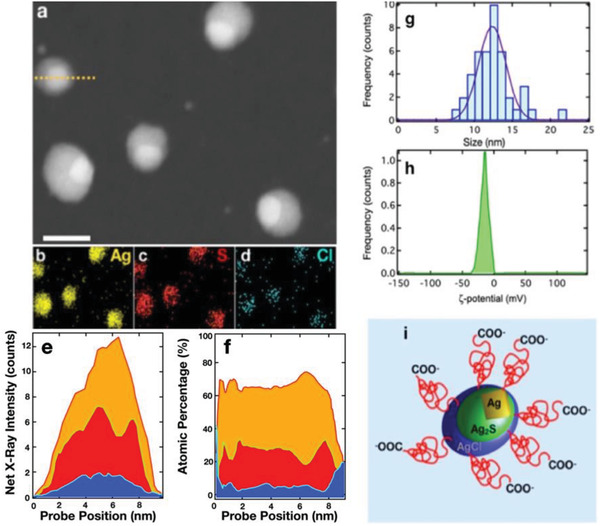
Structure of the superdots. a) HAADF‐STEM micrograph of the synthetized superdots. Scale bar is 10 nm. b) 2D EDS mapping of the spatial distribution of Ag, c) S, and d) Cl atoms. e) Net X‐ray intensity profiles and f atomic percentage obtained from the dashed yellow line marked in (a), note how the Ag/S ratio increases at the edges of the dots, which coincides with the electrodense area. g) Size distribution of the superdots. h) *z*‐potential distribution of PEG superdots in aqueous solution. i) Schematic representation of the superdots covered with PEG carboxyl terminal.

The optical agents used along this work were the recently fabricated Ag_2_S superdots. A complete description of their synthesis and characterization can be found elsewhere.^[^ ^26^ ^]^ These nanoparticles consist of Ag/Ag_2_S cores covered by an AgCl shell that reduces the surface‐related nonradiative decays and that increases their quantum yield up to 10%. **Figure** **1**a depicts a high‐angle annular dark‐field scanning transmission electron micrograph (HAADF‐STEM) of Ag_2_S superdots. Here, we can observe that these nanoparticles exhibit an elliptical shape, with two well‐differentiated regions. EDS elemental mapping obtained from Figure 1a unveils that the more electrodense regions match with Ag‐enriched areas as seen in Figure 1b, while the lower electrodense parts fit with the S‐enriched ones, as shown in Figure 1c. Also, Cl atoms are homogenously distributed around the nanoparticles as seen in Figure 1d. The anisotropic distribution of the elements within the nanoparticles is observed in the net X‐ray intensity profile shown in Figure 1e. Here, we can observe that the maximum of the X‐ray intensity assigned Ag and S atoms arises from the electrodense core and the outer area respectively. The presence of Cl atoms is related to the presence of a AgCl shell as discussed elsewhere.^[^ ^26^ ^]^ The Ag_2_S superdots exhibit a mean size of 13 nm as seen in Figure 1f. To provide colloidal stability in aqueous solution, the superdots were functionalized with a bifunctional HS‐PEG‐COOH, that yields negative charged nanoparticles with a mean value of ‐23 mV, as seen Figure 1g. Figure 1h represents schematically the structure of the synthesized superdots.

**Figure 2:**
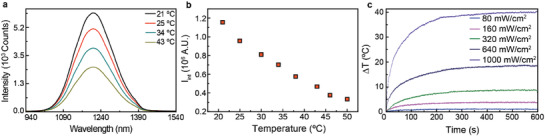
Multifunctionality of Ag_2_S superdots as optical agents. a) Thermal dependence of luminescence spectra. b) Calibration curve of integrated intensity. c) Temperature increment as observed after exciting a colloidal suspension of Ag_2_S superdots under different excitation power densities. The concentration of the nanoparticles was 0.8 mg mL^−1^.

Under 808 nm light excitation, the optimal wavelength for in vivo imaging experiments considering the minimization of heating effects, ^[^ ^27^, ^28^, ^29^ ^]^ they present a thermally dependent broad emission centred at 1210 nm (**Figure** **2**a). Among the different parameters that could be used for thermal reading, the integrated intensity, *I*_int_, presents itself as the most straightforward. After all, the use of this parameter would allow the monitoring of thermal dynamics even in cases where the employed detectors are deprived of spectral resolution (such as InGaAs infrared cameras). Figure 2b shows the temperature dependence of *I*_int_. From the analysis of the experimental data, an intensity‐based relative thermal sensitivity of 3.9% °C^–1^ was estimated at 20 °C. This is, indeed, very similar to the intensity based thermal sensitivity reported for conventional Ag_2_S dots.^[^ ^30^, ^31^ ^]^ At this stage, it is important to point out that, although the emitted intensity is not suited for absolute temperature estimations,^[^ ^24^ ^]^ it is still capable of providing some level of information about the time‐dependent part of the cooling dynamics. This, in turn, is sufficient for tissue diagnosis.

The potential of Ag_2_S superdots as photothermal agents was demonstrated through an experiment in which a colloidal suspension of Ag_2_S superdots was illuminated with an 808 nm laser at different power densities, *I*. The temperature of the solution was then dynamically recorded by a thermographic camera (Figure 2c). As observed, for a laser power density of 1W cm^−2^, typical in in vivo photothermal therapies of tumors, the Ag_2_S superdots could increase the solution temperature by more than 40 ⁰C. This, in turn, reveals that the Ag_2_S superdots possess a non‐negligible laser‐to‐heat conversion efficiency. The steady‐state temperature increment was found to be proportional to the laser power density. At room temperature, the Quantum Yield (QY) of superdots in PBS has been determined to be as low as 10%, indicating that the radiative decay rate is much smaller than the nonradiative ones. The heating efficiency of Ag_2_S NPs (*η*_h_) is defined as the fraction of the absorbed energy that it is released in the form of heat (Section S1):

(1) $$\eta_{h}=\frac{\mathrm{heat}\;\mathrm{released}}{\mathrm{absorbed}\;\mathrm{optical}\;\mathrm{energy}}\;=\;1-QY\frac{\lambda_{p}}{\lambda_{f}}$$

where *λ*_p_ = 808 nm and *λ*_f_ = 1200 nm are the excitation and averaged fluorescence wavelengths, respectively. Thus, according to Equation (1) the photothermal conversion efficiency of Ag_2_S superdots is around 90%. This is in accordance with the relevant heating observed in a solution of Ag_2_S superdots (Figure 2c). Due to the capacity of Ag_2_S superdots for simultaneous heating and thermal sensing, they emerge as excellent candidates for TTh. The estimation of the light‐to‐heat conversion efficiency based on equation (1) assumes that all the nonradiative decays contribute to local heating that is, indeed, a first order approach. Independently on the exact value provided by equation (1) to the light‐to‐heat conversion efficiency we would like to note that this expression just reveals that the low Quantum Yield of the superdots used in this work ensure an efficient light‐to‐heat conversion efficiency.

### 2.2 In Vivo Transient Thermometry Experiments

**Figure 3:**
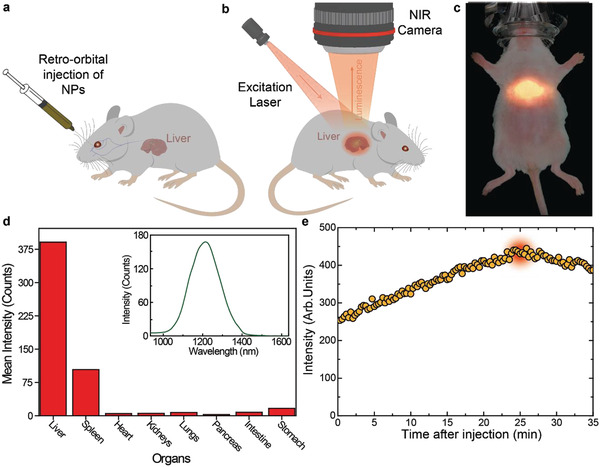
In vivo transient thermometry. Schematic representation of a) retro‐orbital injection of Ag_2_S superdots in a CD1 female mouse and b) experimental setup used for the recording of the luminescence intensity. c) Near‐infrared luminescence image of a characteristic individual as obtained immediately after the injection of Ag_2_S superdots. d) Biodistribution of the Ag_2_S superdots in the different organs of a CD1 mouse after being intravenously injected with them. e) Time course of the NIR‐II intensity generated from the liver of a CD1 mouse after intravenous injection of Ag_2_S superdots.

The experimental procedure here adopted for in vivo evaluation of liver thermal dynamics is summarized in **Figure** **3**. CD1 female mice (n = 6) were anesthetized with inhaled isoflurane and subjected to a retro‐orbital injection of 100 µl of a 1.5 mg mL^−1^ solution of Ag_2_S superdots in PBS (Figure 3a). To check their accumulation into the liver, the animal was placed inside an imaging chamber and illuminated with an 808 nm laser fiber (power density of 50 mW cm^−2^). The spot diameter was wide enough to illuminate the abdominal region corresponding to the anatomical location of the liver. The NIR fluorescence image of the mouse was recorded by a thermoelectrically cooled InGaAs camera (Figure 3b), and significant emission was then detected (Figure 3c）. Ex vivo hyperspectral analysis of all the organs revealed that most of Ag_2_S superdots were preferentially accumulated at liver and spleen (see Figure 3d). As a final caution before the TTh measurements, the time‐tracking of the NIR luminescence was recorded and it revealed a maximum intensity at the liver 25 min after injection (Figure 3e). Thus, all the measurements were conducted 25 min after injection. This not only ensured a larger fluorescence signal but also a minimal variation of luminescence with time.

**Figure 4:**
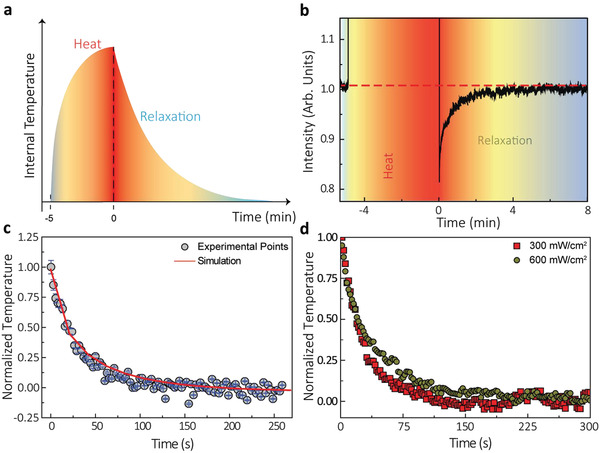
Dependence of relaxation profiles with the state of the liver. a) Working principle of transient thermometry. b) Time evolution of the luminescence intensity during the heating and cooling cycles. c) Normalized thermal transient as experimentally obtained (grey circles) and numerically simulated (red line). d) Representative thermal transients obtained after changing the excitation power density during the heating cycle. The log‐scale was used to evidence the differences between the curves. The luminescence intensity was recorded under the same conditions for five different mice: exposure time of 0.5 s and transients recorded btween the 22nd and the 27th min after intravenous injection. The dynamics of the luminescence intensity was calculated with the mean intensity of the illuminated area. Error bars correspond to the standard deviation of the mean.

For TTh, the diameter spot of the 808 nm laser was reduced to 0.3 cm (which accounted for approximately 10% of the top surface of the murine liver) so that no collateral damage could be induced in the surrounding organs. Its power, on the other hand, was correspondingly adjusted so that the light reaching the liver was enough to activate the nanoheaters inside of it (ranging from 100 up to 900 mW cm^−2^). A temperature increment was then induced, and the steady state was achieved 5 min after illumination (**Figure** **4**a). Immediately after this, the excitation power density was reduced to 50 mW cm^−2^ to record the intensity variation in the absence of significant heating. Figure 4a includes a representative time evolution of the luminescence intensity generated by the Ag_2_S superdots at the liver during a TTh experiment. In the heating cycle, the fluorescence signal saturates due to the high‐power density utilized. Immediately after switching off the heating beam, the NIR fluorescence signal generated at the liver was found to be below its initial value. This indicates effective heating of the liver (remind Figure 2a i.e., fluorescence intensity decreases with temperature). As the liver recovers its basal temperature the fluorescence intensity gradually recovers its initial value. This recovery of luminescence signal not only guarantees that the initial temperature has been recovered but also that NPs degradation are neglectful in the time scale of our measurements. Nevertheless, it is true that transient thermometry, in broader terms, could be severely affected by the formation of protein corona that could lead to brightness alterations not related to thermal processes. Future in vivo long‐term experiments will be necessary to properly unravel potential biological parameters affecting the absolute intensity signal provided by nanoparticles.

If the spectral distortions induced by the layers of tissue between the liver and the surface of the abdomen were negligible, the relation between the luminescence intensity and the temperature could be simply computed through the calibration included in Figure 2b. Nevertheless, as previous works have shown, such an approach is not valid.^[^ ^24^ ^]^ To check the validity of considering the intensity profile as a thermal profile we need first compare the experimental results to theoretical predictions. The time evolution of liver temperature during thermal relaxation Δ*T*_liver_(*t*), can be properly described by (Section S2):

(2) $$\Delta T_{\mathrm{liver}}\;\left(t\right)=\;\Delta T_{\max}\cdot \exp\left(-{\left(\frac{t}{\tau_{c}}\right)}^{\beta }\right)$$

where Δ*T*_max_ is the temperature increment induced in the heating cycle, *τ*_c_ is a parameter that depends primarily on the thermal properties of the tissues found between the skin and the internal organ, and *β* is a parameter that depends primarily on the location of the thermometer within the liver. Expression (2) is known as the stretched exponential function. Their main advantages over the use of a single‐exponential decay (as utilized in previous works)^[^ ^21^, ^22^ ^]^ are: (i) it is more realistic as it considers that distinct layers of tissues will have different levels of importance in the heat distribution, and (ii) it can fit relaxation profiles obtained under various circumstances (including the ones where a single‐exponential is expected, i.e., *β* = 1). Hence, as opposed to previous works, the characteristic relaxation time (*τ*_relax_) cannot be defined as the moment in which the normalized Δ*T*_liver_ signal reduces from 1 to 1/e. Instead, according to literature, it has to be defined as the area below the normalized curve (specifically, *τ*_relax_ = *τ*_c_ *β* ^−1^ Γ(*β* ^−1^)).^[^ ^32^, ^33^ ^]^

Remarkably, after taking the luminescence generated by the nanoparticles located in the liver and normalizing it, the decay included in Figure 4c is found to follow the profile of Equation (2) Experimental data fits nicely to a stretched exponential (R^2^ ≈ 0.99). In addition, the fitting provides us a relaxation time for a healthy liver in absence of relevant thermal loading of 27 ± 3 s. This value is in excellent agreement with that provided by in silico simulations (red curve in Figure 4c when considering the thermal relaxation of a liver under the same experimental conditions (for details on the thermal properties utilized and in silico simulations, see Methods). These two facts together suggest that, though the absolute value of intensity could be affected by the presence of the tissue, the time‐evolution of intensity well reproduces the thermal dynamics of the liver (even in the presence of tissue distortions). If that were not the case, either the trend of the relaxation or the value of the characteristic relaxation time should significantly differ from what it is theoretically and numerically expected. Considering the latest results questioning its reliability, such inference shows at least an improvement in the potential of luminescence thermometry. After all, even if the luminescence thermometers are not capable of providing the absolute values of temperature or temperature increments, they can still provide information on the thermal properties of the tissue through the evaluation of their time‐dependent part of the luminescence dynamics.

Figure 4d includes the in vivo normalized liver cooling curves obtained after heating cycles produced under 300 and 650 mW cm^−2^ power densities. These two values were chosen to represent the cases of (i) a slightly higher power density than the one with no damaging heat and (ii) a mildly higher power density. Clear differences were observed. While the liver subjected to 300 mW cm^−2^ presented virtually the same *τ*_relax_ as the one obtained under 200 mW cm^−2^, the liver subjected to 650 mW cm^−2^ showed slower cooling dynamics. Indeed, a characteristic relaxation time of *τ*_relax_ = 42 s was found. In other words, the mild change in the excitation power caused an increment of approximately 45% in the liver relaxation time. The reason for this is found in the fact that a higher laser power density leads to an increment in the liver temperature. This, in turn, results in an observable change in the liver relaxation time. Therefore, *τ*_relax_ emerges as a viable thermometric parameter for experiments. The link between *τ*_relax_ and liver temperature could arise from the temperature dependence of tissue properties,^[^ ^34^, ^35^ ^]^ and/or on the influence of starting temperature on the cooling dynamics. Given the right calibration, the value of *τ*_relax_ could be inversed to provide the value of liver temperature increment (Δ*T*_liver_). Under such conditions, luminescence thermometry would then go back to its initially proposed potential.

**Figure 5:**
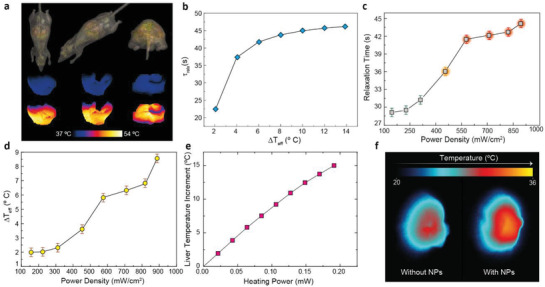
In vivo and in silico observation of liver temperature in a mouse. a) (Top) Different views of the mouse virtual model used in the numerical simulations. Numerical thermal images of the liver at low (Middle) and high (Bottom) temperatures. b) Dependence of the characteristic relaxation time with the effective temperature increment induced by the nanoparticles. c) Experimental values obtained for the characteristic relaxation time under different excitation power densities. d) Temperature variation inside the liver as calculated through the calibration obtained from the numerical simulations. e) Dependence of the in vivo liver temperature increment with the heating power applied as obtained through in silico simulations. f) Thermal images of the liver obtained through luminescence transient thermometry.

Determining the dependence of *τ*_relax_ with the liver temperature increment is not an easy task: *τ*_relax_ depends on (i) the layers of tissue between the thermometers and the detection system and (ii) all the biological processes involving heat distribution (such as blood flow‐induced dissipation). To investigate the relation between *τ*_relax_ and Δ*T*_liver_ we performed numerical simulations of the heat diffusion in a computable phantom of a normal male mouse (featuring 43 different types of tissues, **Figure** **5**a. In these simulations we considered the thermal dependence of physical properties of the liver as previously reported in the literature.^[^ ^36^ ^]^ While the physical and thermal properties of the surrounding tissues were the ones included in the IT'IS Foundation database,^[^ ^37^ ^]^ the physical properties of the liver were modified according to Manago et al.^[^ ^38^ ^]^ More details can be found in Section S3 of Supporting Information. Figure 5b shows the dependence of *τ*_relax_ as a function of the total variation of temperature during cooling (Δ*T*_liver_). As observed, though not linear, there is a unique correspondence between the numerically obtained *τ*_relax_ and Δ*T*_liver_.

Data of Figure 5b therefore, opens the way to get an absolute reading of liver temperature through the experimental determination of *τ*_relax_. In the present case, we have tried to apply it for the determination of the thermal loading of the liver during a photothermal process as a function of laser power intensity. For doing so we have experimentally determined the variation of *τ*_relax_ as a function of the heating laser power density. All the measurements were performed in the same experimental conditions set for Figure 4. Results are included in Figure 5c Three regimens were observed: (i) for power densities below 300 mW cm^−2^ the liver relaxation time remained virtually constant and close to the value reported in Figure 4c. (ii) from 300 to 600 mW cm^−2^ the liver cooling time was found to increase from 31 to 42 s; (iii) above 600 mW cm^−2^, the liver relaxation time remained constant at 43 s. To convert these relaxation times into temperature units, the data of Figure 5b (provided by in silico simulations) were used. Results are shown in Figure 5c and they reveal, as expected, a monotonous increment of liver temperature with the 808 nm laser power density. Data of Figure 5c provide, in a first order approximation, a net radiation‐to‐heat conversion rate of 10 °C⋅cm^2^ W^−1^. This is significantly lower than the heating rate obtained for the colloidal suspension of Ag_2_S superdots in PBS (40 °C⋅cm^2^ W^−1^ as obtained from Figure 2c. This, in turn, can be explained by two different effects:

i) The presence of the tissues between the liver and the detection system causes the excitation light to be attenuated. The tissue thickness between skin and liver has been estimated to be approximately 5 mm. Considering that the average extinction coefficient for murine skin tissues at 808 nm can be assumed to be 13 cm^–1^, the Beer‐Lambert Law predicts that only 0.15% of the incident 808 nm radiation reaches the liver.^[^ ^39^ ^]^ This means, for instance, that for the maximum incident pump density used in this work (0.9 W cm^−2^) tissue extinction reduces the 808 nm laser power density reaching the liver down to 1.35·10^–3^ W cm^−2^. Assuming a spot diameter of 0.3 cm, the total laser power reaching the liver is estimated to be only 0.1 mW.
ii) The actual concentration of nanoparticles in the liver differs from that of the injected solution due to the natural biodistribution of Ag_2_S superdots.

While (i) causes a decrease in the delivered energy to the injected nanoparticles, (ii) implies on a reduced number of nanoheaters inside the organs. Both effects result in a reduction of the heat generated by the injected nanoparticles. With no surprise, accounting for them is not an easy task. But, once again, in silico simulations can shed some light. We have performed numerical simulations to register the in vivo liver temperature as a function of the heating power. Results are included in Figure 5e. Simulations indicate that to achieve the largest liver heating obtained in this work (8.5 ⁰C obtained for the maximum laser power density of 0.9 W cm^−2^) the total heating power provided to the liver is close to 0.09 mW. This is in a very good agreement with the 808 nm laser power experimentally determined to reach the liver (calculated above to be 0.1 mW). This, in turn, means that almost all the radiation reaching the liver is being absorbed and converted into heat. To have a general idea of the role played by Ag_2_S superdots in this conversion, a simple experiment was conceived. In it, an ex vivo liver of a mouse previously injected with the NPs was exposed to 808 nm illumination and its temperature achieved in the steady‐stated was compared with the one of an ex vivo liver of a mouse which had not been injected with Ag_2_S superdots. The results indicated that most of the absorption was caused by the tissue itself (see Figure 5f. In fact, if the relative difference between the maximum temperature increment reached in the livers can be used as an indicator of the relative contribution of the NPs to the overall heating, the data suggest that NPs are only responsible for approximately 30% of the net heating at high laser power densities). This, in turn, reveals photothermal therapies as low efficiency treatments if the safety of the surrounding tissues is something to be controlled with the limits imposed on the radiation dose (< 1.5 W cm^−2^).

**Figure 6:**
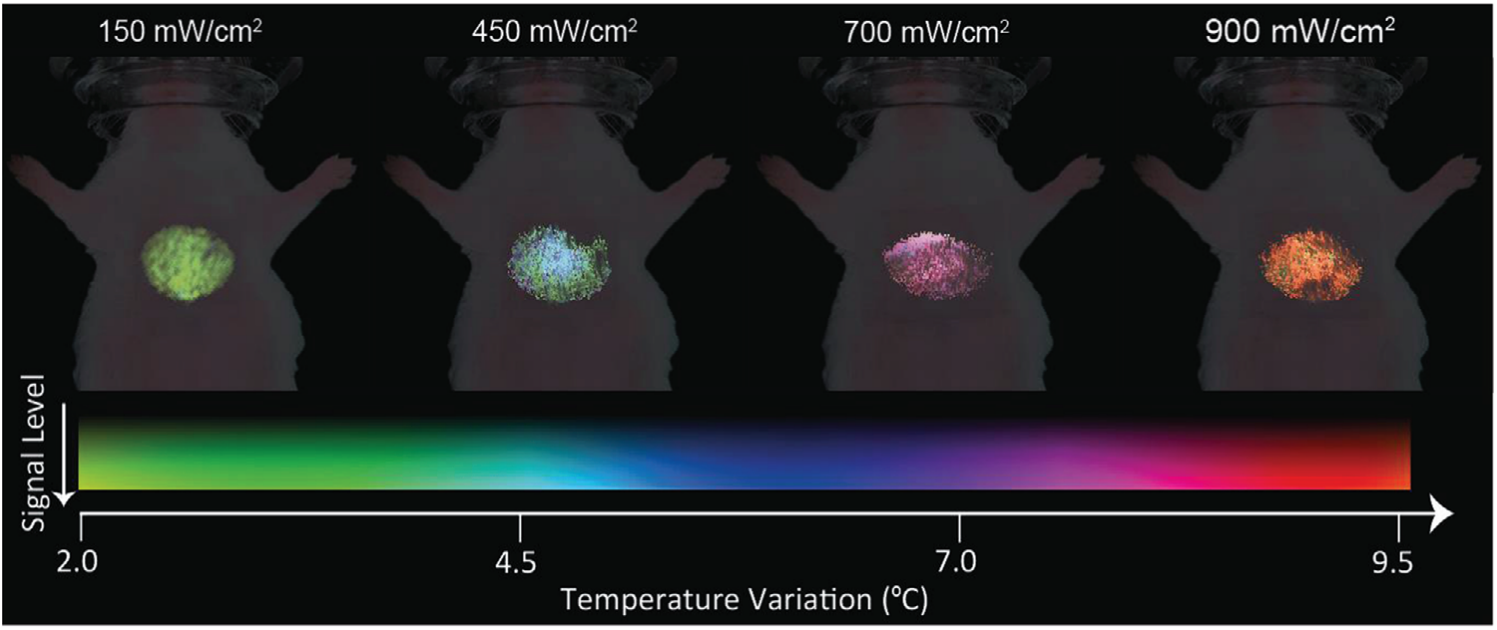
Thermal images of the liver as obtained through luminescence transient thermometry. Each pixel has been fitted to a stretched exponential and the resulting relaxation times were computed.

As the performed analysis was based on the acquisition of luminescence images at various times after the end of the heating cycle, the results here reported also present the potential of thermal imaging. After all, each pixel in the field of view can have its intensity evolution fitted to the stretched exponential. This function, in turn, is of particular interest from the computational point of view and finds applicability in areas such as fluorescence lifetime imaging (FLIM) where spatial discrimination of lifetimes is used to distinguish regions (or features) of a system.^[^ ^33^, ^40^, ^41^ ^]^ Similarly, one could apply such a concept to the thermal relaxation time and build a map (2D figure) that, by itself,would completely characterize the thermal dynamics of the internal organ. In other words, after analysing the set of figures obtained after the heating cycle, a new figure, whose pixels’ intensities would correspond to the local temperature, would be produced. To achieve this goal, a code was written and is described in Section S4. After repeating the process for the data obtained in the last experiment and applying a suitable pseudo‐colour scale, one will encounter the images included in **Figure** **6**. The transition from green to red indicates the temperature increment. The results agree quite well with the values provided in Figure 6 even though the latter were computed with the mean intensity of the illuminated region and the former results from a pixel‐by‐pixel analysis. Figure 6 constitutes the first thermal images reported at the in vivo level for an internal organ and demonstrate the great potential of the synergy between in vivo and in silico predictions to achieve contactless thermal information of internal organs.

### 2.3 Justification for the Sensitivity of TTh Based on In Silico Experiments

The results described in previous sections demonstrate the potential of TTh for contactless monitoring of the liver temperature after a heating cycle, but it provides no information about the nature of the heat‐induced modifications. To further understand it, one needs to take into account the equations describing the thermal dependence of the properties of tissues. On the one hand, it has been established that neither the thermal conductivity nor the heat capacity undergo noticeable changes within the considered temperature range for healthy liver.^[^ ^42^, ^43^ ^]^ Therefore, these parameters cannot account for the relaxation changes experimentally seen. On the other hand, looking into other relevant parameters, it is known that *ω* increases the heating time needed to produce some temperature elevation in organs, as well as decreases the initial time required to cool them down that leads in turn to a monotonic stretch towards thermal equilibrium.^[^ ^44^ ^]^ At the maximum temperatures achieved in our heating cycles and the corresponding exposure times, protein structural changes, hydrogen bonding breaking and some tissue retraction can take place.^[^ ^45^ ^]^ The occurrence of oedema is also possible due to vasodilatory pooling, and in fact some signs of it are observed in post‐mortem optical images from the illuminated liver (Section S5). In these conditions, the simulations suggest that blood perfusion becomes defective in certain liver portions and thermal relaxation proceeds differently. More precisely, the damping effect of *ω* during cooling is modified so that temperature decays more slowly at the beginning and then struggles to equilibrate. This, in turn, provides a justification, at the fundamental level, for the sensitivity of the technique.

## 3 Conclusion

In summary, we have shown that luminescent nanothermometers can be used in small animal models to infer the temperature variations in an internal organ at the 2D level. In particular, infrared emitting Ag_2_S superdots were capable of determining the thermal dynamics of a liver under in vivo conditions by highly penetrating transient thermometry. Additionally, when activating their light‐to‐heat conversion capability, we were able to observe variations in the characteristic relaxation time of the livers after intense heating cycles. Despite the attenuation of light into the surrounding tissues, these variations could be correlated with the temperature increment induced by Ag_2_S dots. After comparing the in vivo results with the heating efficiency of Ag_2_S superdots obtained under normal conditions, the potential of photothermal treatment was put into question. Numerical simulations involving the heat diffusion in mouse computable phantom determined that the increase in the relaxation time of a liver could be explained by the permanent change of blood perfusion after applying relatively long thermal gradients inside the organ. Thus, the present work provides a timely and novel kind of contribution in terms of the potential of NIR luminescent nanothermometers as tools for the control of thermal damage induced by optically activated nanoheaters.

## 4 Experimental Section

##### In Silico Model of a Mouse—Thermal Properties

Numerical simulations have been performed in a realistic computer mouse model using a transient solver that employs the finite‐difference time‐domain (FDTD) method, allowing for a better local resolution. The mouse model is available through the IT'IS FOUNDATION as part of the Virtual Family computational models.^[^ ^46^ ^]^ In order to mimic the in vivo setup as much as possible, the posture of the mouse virtual model has been adapted using a physics‐based poser engine included in the Sim4Life platform.^[^ ^47^ ^]^ The employed thermoregulation model is based on the Pennes bioheat equation (Eq. S1) containing a lumped term embodying *ω*_b_, *ρ*_b_,  *c*_b_ and *T*_b_ (representing, respectively, the perfusion rate, the density, the specific heat capacity, and the temperature of the blood.) to account for heat carried away by blood perfusion, or heat transfer rate term.

Also, in all the simulations the model has undergone a discretization into cubical voxels of the whole computational domain using a homogeneous grid with 0.3 mm x 0.3 mm x 0.3 mm resolution. The system has been evaluated for each tissue taking into account the corresponding tissue physical properties (**Table** **1**. In addition, only the voxels with a value within a narrow band defined by some pre‐defined limits around transition temperatures are tested after each iteration and reassigned to other segments of the temperature dependence, as described in literature.^[^ ^48^ ^]^ The width of the band considered here is 0.5 °C above and below the corresponding temperature limits.

**Table 1:** Relevant physical parameters of the tissues present in the thermal simulations. Source: reference^[^ ^33^ ^]^

Tissue	Density [kg m^−^³]	Heat Capacity [J kg^−1^ °C^−1^]	Thermal Conductivity [W m^−1^ °C^−1^]	Heat Transfer Rate [mL min^−1^ kg^−1^]	Heat Generation Rate [W kg^−1^]
Adrenal Gland	1028	3513	0.44	1458	22.58
Bile	928	4037	0.58	0	0
Bone (Cortical)	1908	1313	0.32	10	0.15
Fat	911	2348	0.21	33	0.51
Gallbladder	1071	3716	0.52	30	0.46
Heart Muscle	1081	3686	0.56	1026	39.45
Kidney	1066	3763	0.53	3795	18.05
Liver	1079^a)^	3540	0.52	860	9.93
Lung	394	3886	0.39	401	6.21
Pancreas	1087	3164	0.51	767	11.89
Skin	1109	3391	0.37	106	1.65
Small Intestine	1030	3595	0.49	1026	15.89
Spleen	1089	3596	0.53	1557	24.11
Stomach	1088	3690	0.53	460	7.13

^a)^To account for the variation of density associated to the use of different animals and the tissue response to the irradiation, the values of liver density used for each has been chosen from within a range determined by the following average, minimum, maximum and standard deviation values, respectively: 1079, 1050, 1158, ±53. This special attention has been paid to the liver, which is the mainly affected organ, while this variation has been neglected in the case of the other tissues after some preliminary testing.

The possible extent of the temperature variation of the most relevant physical properties has been also considered in the simulations. On the one hand, it has been established that neither *κ* nor *c* undergo noticeable changes within the considered temperature range for healthy liver;^[^ ^42^, ^43^ ^]^ thus, these parameters cannot account for the relaxation changes seen experimentally. On the other hand, looking into other relevant parameters, it is known that *ω* increases the heating time needed to produce some temperature elevation in organs, as well as decreases the initial time required to cool them down that leads in turn to a monotonic stretch towards thermal equilibrium.^[^ ^44^ ^]^ However, this behavior only applies to undamaged tissues. At the maximum temperatures achieved in the PTT and the corresponding exposure times, protein structural changes, hydrogen bonding breaking and some tissue retraction may take place.^[^ ^45^ ^]^ The occurrence of oedema is also possible due to vasodilatory pooling, and in fact some subtle signs of it are observed in post‐mortem optical images from the treated liver (Figure S3, Supporting Information). The extent of this area is proportional to the volume of damaged tissue, which has been experimentally estimated to be 10%, helps in estimating the damage extension. In these conditions, blood perfusion becomes defective in wounded liver portions and thermal relaxation proceeds differently after PTT. More precisely, the damping effect of w during cooling is modified so that temperature decays slower at the beginning and then struggles to equilibrate. Due to this induced dysfunction, blood perfusion was assumed constant in all the simulations. Moreover, the observed variation against a piecewise linear approach (see section S3 in Supplementary Information) where the perfusion changes with the temperature, is less than 1% in the organ of interest.

Regarding the relaxation times to temperature relationship (Figure 5b, 7 transient simulations along 300 s using IT'IS foundation database^[^ ^37^ ^]^ for all tissues except the portion of liver radiated which corresponds to the 10% of the total organ volume and was assumed to have a reduced portion of water content in this area. This region was modelled as a shrunk sphere in the middle part of the liver. All tissues except the liver were set to the basal temperature seen in the in vivo experiments (34°C). The initial temperature for the liver and its radiated portion was determined calculating the absolute value for each Δ*T* shown in the graph. In the particular case of the data plotted in Figure 5e three steady‐state simulations using IT'IS foundation database for all tissues^[^ ^37^ ^]^ have been carried out, assuming a laser power high enough to produce an increment of the temperature in the liver of 6, 12, and 15°C (**Table** **2**).

**Table 2:** Radiation power values considered to produce distinct temperature increments in the liver

Δ*T* [°C]	Power density [W m^−3^]	Radiation power [Mw]
6	0.527E6	0.07
12	1.128E6	0.14
15	1.529E6	0.19

##### In Vivo Experiments

In vivo experiments were approved by the regional authority for animal experimentation of Comunidad de Madrid and were conducted in agreement with the Universidad Autónoma de Madrid (UAM) Ethics Committee, in compliance with the European Union directives 63/2010UE and Spanish regulation RD 53/2013. Experiments were designed in order to use the minimal amount of animals, in accordance with the 3Rs ethical principle. No randomization or blind studies were performed.

For this study, six CD1 female mice (8–14 weeks old, weighing 25–39 g) bred at the animal facility at UAM were used. Mice were anesthetized prior to the imaging experiments in an induction chamber with a continuous flow of 4% isoflurane (Forane, AbbVie Spain, S.L.U) in 0.5 mL min^−1^ of 100% oxygen until loss of righting reflex was confirmed and breathing rhythm was significantly slowed. Anesthesia was maintained throughout the experiments by means of facemask inhalation of 1.5% isoflurane and core body temperature was kept at 36 ± 1°C, as measured with a rectal probe, using a heating pad. To have a higher temperature increment in the liver while keeping the safety conditions on laser power, the mouse core temperature was decreased down to 34 °C through the use of a heating pad set at 32 °C, and this was monitored with a rectal probe. After keeping this temperature constant for 5 min, we induced a heating cycle on the liver with a laser fiber coupled. Preliminary studies were performed to determine the optimal power densities that should be used to achieve a significant temperature. Once the steady state was achieved (typically after 2 or 3 min), the laser power then kept at the same value to ensure that the heat distribution was taking place in the organ for 5 min. After this, the laser power density was adjusted so that no additional heating was produced, and the thermal relaxation could be recorded. External temperature was monitored with an infrared thermographic camera.

##### Statistical Analysis

Five different mice have been used for the analysis of the average value of the characteristic relaxation time. The error was estimated through the calculation of the standard deviation of the mean. For the study of the dependence of the thermal relaxation time with the excitation power density, the sample size was reduced to one in order to minimize animal suffering. The error in their calculation, on the other hand, was computed through the error of the fitting function. The software utilized for analysis was OriginLab.

## Conflict of Interest

The authors declare no conflict of interest.

## Supporting information

Supporting Information

## Data Availability

The data that support the findings of this study are available from the corresponding author upon reasonable request.
