# Supplementary material for: Reaching Deeper: Absolute In Vivo Thermal Reading of Liver by Combining Superbright Ag2S Nanothermometers and In Silico Simulations
Source: Adv Sci (Weinh). 2021 Mar 3;8(9):2003838. doi: 10.1002/advs.202003838 (PMC8097345; doi:10.1002/advs.202003838)
Supplement: Supplementary file 1 — Supporting Information [file ADVS-8-2003838-s001.pdf]

© 2021 Wiley-VCH GmbH

ADVANCED SCIENCE Open Access

Supporting Information

for *Adv. Sci.,* DOI: 10.1002/advs.202003838

Reaching Deeper: Absolute *In Vivo* Thermal Reading of Liver by Combining Super-bright Ag2S Nanothermometers and *In Silico* Simulations

*José Lifante, Yingli Shen, Irene Zabala-Gutierrez, Irene Rubia, Daniel Ortega, Nuria Fernandez, Sonia Melle, Miriam Granado, Jorge Rubio-Retama, Daniel Jaque, and Erving Ximendes**

Supporting Information

Reaching deeper: absolute *in vivo* thermal reading of liver by combining super-bright Ag2S nanothermometers and *in silico* simulations

*José Lifante, Yingli Shen, Irene Zabala-Gutierrez, Irene Rubia, Daniel Ortega, Nuria Fernandez, Sonia Melle, Miriam Granado, Jorge Rubio-Retama, Daniel Jaque, and Erving Ximendes**

Section S1 – Deduction of the fraction of absorbed energy released as heat.

Section S2 - Heat conduction in a composite biomaterial with perfusion.

Section S3 - Thermal dependence of physical properties of murine tissues.

Section S4 - Algorithm for relaxation time imaging

Section S5 – Postmortem optical images of livers

Section S6 - References

Section S1 - Deduction of the fraction of absorbed energy released as heat

The radiation-to-heat conversion efficiency (η_h) of the quantum dots (QDs) could be defined as the fraction of the absorbed pump power that it is transformed into heat by the Ag_2S NPs. The fluorescence Quantum Yield (QY) is defined as the radiative decay probability divided y the total decay probability. Those magnitudes are related not only in the case of semiconductor nanocrystals but also in any luminescence system.[S2,S3] When an excitation photon with a wavelength λ_p is absorbed by a Ag_2S NP the incoming energy of this event is $E_{IN} = \frac{hc}{\lambda_p}$. We assume that all the absorbed photons create an electron in the conduction band, i.e. we assume that the pump quantum efficiency is η_p = 100%. Once the electron generated by the absorption of the excitation photon is created it decays radiatively (emitting heat) to the bottom of the conduction band. In this process the energy delivered in the form of heat is $E_{CB} = hc\left(\frac{1}{\lambda_p} - \frac{1}{\lambda_F}\right)$. Once at the bottom of the conduction band, it could lead to the emission of a fluorescence photon of energy $E_{OUT} = \frac{hc}{\lambda_F}$ where λ_F is the averaged wavelength of the fluorescence spectrum ($\lambda_F \approx$ 1200 nm in our case). The emitted energy in the form of photon emission would be the number of radiative decays times the energy of each decay ($\frac{hc}{\lambda_F}$) so that the radiative emission rate can be written as: $R_{rad}^{energy} = W_{rad} \cdot \frac{hc}{\lambda_F}$. In the same way, the heat generation rate due to non radiative transitions, i.e. de-excitations delivering heat, from the bottom of the conduction band to the valence band would be given by $R_{non-rad}^{energy} = W_{non-rad} \cdot \frac{hc}{\lambda_F}$. The total energy delivered to the system is the excitation rate (R_{pump}) times the energy of the excitation photon ($\frac{hc}{\lambda_p}$). In a steady state situation, the electron density at the conduction bands should be constant so that the excitation rate should be the same as the total de-excitation rate so that $R_{pump} = W_{tot}$.In this conditions, we have that energy absoprtion rate is given by the $R_{abs}^{energy} = W_{tot} \cdot \frac{hc}{\lambda_p}$ and that the heat generation due to the non-radiative

decay of the electrons within the conduction band is $R_{CB}^{energy} = W_{tot} \cdot hc\left(\frac{1}{\lambda_p} - \frac{1}{\lambda_F}\right)$. The total rate of heat generation is then given by:

$$R_{heat}^{energy} = R_{CB}^{energy} + R_{non-rad}^{energy} = W_{tot} \cdot hc\left(\frac{1}{\lambda_p} - \frac{1}{\lambda_F}\right) + W_{non-rad} \cdot \frac{hc}{\lambda_F} \quad \text{(Equation S1)}$$

The rate of absorbed energy is $R_{abs}^{energy} = W_{tot} \cdot \frac{hc}{\lambda_p}$ so that we can write the heating efficiency as:

$$\eta_h = \frac{R_{heat}^{energy}}{R_{abs}^{energy}} = \frac{W_{tot} \cdot hc\left(\frac{1}{\lambda_p} - \frac{1}{\lambda_F}\right) + W_{non-rad} \cdot \frac{hc}{\lambda_F}}{W_{tot} \cdot \frac{hc}{\lambda_p}} \quad \text{(Equation S2)}$$

This can be written as:

$$\eta_h = \frac{W_{tot} \cdot \frac{hc}{\lambda_p}}{W_{tot} \cdot \frac{hc}{\lambda_p}} + \frac{\frac{hc}{\lambda_F}[W_{non-rad} - W_{tot}]}{W_{tot} \cdot \frac{hc}{\lambda_p}} = 1 + \frac{-W_{rad}}{W_{tot}} \frac{\lambda_p}{\lambda_F} = 1\text{-QY}\frac{\lambda_p}{\lambda_F} \quad \text{(Equation S3)}$$

where we have used $W_{tot} = W_{rad} + W_{non-rad}$ and $QY = \frac{W_{rad}}{W_{tot}}$.

Section S2 - Heat conduction in a composite biomaterial with perfusion

The one-dimensional problem of conduction of heat into a tissue with blood perfusion can be described by the well-known Penne's bioheat equation:

$$\rho c \frac{\partial T}{\partial t} = \frac{\partial}{\partial x}\left(k \frac{\partial T}{\partial x}\right) - \rho_b c_b \omega (T - T_a) + q \qquad \text{(S4)}$$

where ρ, c, k are the density, specific heat and thermal conductivity of the tissue, ω the is the local blood perfusion rate and q is the local heat generation rate. T_a and T are the blood and tissue temperatures, respectively.

This section describes the solution to S1 corresponding to the system depicted in Figure S5 where a number of 4 composite layers is presented. Each layer has its own physical properties and, as a consequence, the parameters of equation (S4) may experience a discontinuity at the interfaces. The top surface of the first layer (epidermis) is exchanging thermal energy by convection with a flow field (h_o) whose ambient temperature is T_o. On the other hand, the interface between muscle and bone (bottom of layer 4) is assumed to be at constant core body temperature, T_{core}.[1, 2] Additionally, both temperature and heat flux are required to be continuous throughout the tissue.

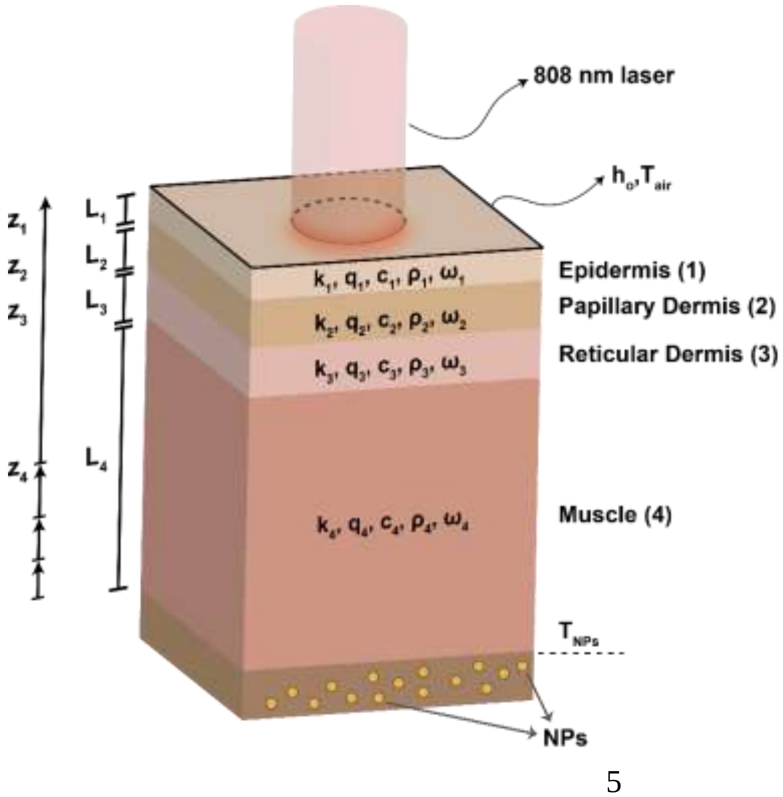

Supplementary Figure S1 – Composite system representation of the perfuse tissue slab

For the sake of simplicity, equation (S1) will be put in a dimensionless form – a common approach recently used in works dealing with the bioheat equation.[3] In order to acomplish that, the spatial domain of the system will be discretized in such a way that each layer will have an associated spatial coordinate that is normalized by its own length (*i.e.*,). Thus, the interface positions may be identified by the coordinates $z_{i-1} = 1$ and $z_i = 0$. The time variable will be scaled by an arbitrary time constant (). Finally, the temperatures of each layer will be described by . Thus, equation (S1) becomes:

$$\frac{1}{\delta_i}\frac{\partial\theta_i}{\partial\tau} + m_i^2\theta_i - \phi_i = \frac{\partial^2\theta_i}{\partial z_i^2} \qquad \text{(S5)}$$

where , and are the dimensionless terms corresponding to perfusion, the constant Fourier number and the volumetric energy source, respectively.

As it was mentioned, the top face of the first layer ($z_1 = 0$) is exposed to convection heat transfer. Therefore, the skin boundary condition is represented as:

$$\frac{\partial\theta_1}{\partial z_1}_{\{z_1=0\}} = H_o(\theta_1(0,\tau) - \Delta\theta_o) \qquad \text{(S6)}$$

where and . On the other hand, the outer end of the last layer is assumed to be at constant body temperature. Thus:

$$\theta_4(1,\tau) = \Delta\theta_L \qquad \text{(S7)}$$

where . The requirement of continuous temperature and heat flux imposes (for i = 2, 3, 4):

$$K_i\frac{\partial\theta_i}{\partial z_i}_{\{z_i=0\}} = K_{i-1}\frac{\partial\theta_{i-1}}{\partial z_{i-1}}_{\{z_{i-1}=1\}} \qquad \text{(S8)}$$

$$\theta_i(0,\tau) = \theta_{i-1}(1,\tau) \quad \text{(S9)}$$

where . Lastly, at $\tau = 0$ the system has some spatially dependent temperature distribution:

$$\theta_i(z_i,0) = F(z_i) \qquad \text{(S10)}$$

S3.1 – Separation of variables

We can suitably construct a solution consisting of a non-homogeneous steady spatially dependent component $\Psi_i(z_i)$ and a position and time dependent homogeneous component $\Theta_i(z_i,\tau)$:[4]

$\theta_i(z_i,\tau) = \Psi_i(z_i) - \Theta_i(z_i,\tau)$ (S11)

In order to make the governing equation and the boundary conditions homogeneous for the $\Theta_i(z_i,\tau)$ component, we impose that $\Psi_i(z_i)$ satisfies the non-homogeneous components of equations S2–S7:

$m_i^2\Psi_i - \phi_i = \Psi_i{}''$ (S12a)

$\Psi_1{}'(0) = H_o(\Psi_1(0) - \Delta\theta_o)$ (S12b)

$\Psi_{\mathrm{N}}(1) = \Delta\theta_L$ (S12c)

$K_i\Psi_i'(0) = K_{i-1}\,\Psi_{i-1}'(1)$ (S12d)

$\Psi_1(0) = \Psi_{i-1}(1)\;\forall\; i = \{2,\dots,N\}$ (S12e)

This, in turns, enables the separation of variables of $\Theta_i(z_i,\tau) = Z_i(z_i) \times \Gamma_i(\tau)$. Thus, substituting equation (S8) in equations S2-S7, we find:

$\frac{1}{\delta_i}\frac{\Gamma_i'(\tau)}{\Gamma_i(\tau)} + m_i^2 = \frac{Z_i''(z_i)}{Z_i(z_i)} = -\mu_i^2$ (S13a)

$Z_1'(0) = H_o Z_1(0)$ (S13b)

$Z_N(1)\Gamma(\tau) = 0$ (S13c)

$K_i Z_i'(0)\Gamma_i(\tau) = K_{i-1}Z_{i-1}'(1)\Gamma_{i-1}(\tau)$ (S13d)

$Z_i(0)\Gamma_i(\tau) = Z_{i-1}(1)\Gamma_{i-1}(\tau)$ (S13e)

$Z_i(z_i)\Gamma_i(0) = \Psi_i(z_i) - F_i(x_i)$ (S13f)

where μ_i is the eigenvalue associated with the i-th layer. The quantity $\mu_i^2 + m_i^2$ is anticipated to be a positive real number so that the transient component solutions are of the form:

$\Gamma_i(\tau) = exp(-\delta_i(\mu_i^2 + m_i^2)\tau)$ (S14a)

$Z_i(z_i) = a_i\sin(\mu_i x_i) + b_i\cos(\mu_i x_i)$ (S15a)

where a_i and b_i are integration constants.

S3. 2 – Algorithmic relations

A similar problem containing the conditions described by Equations S13a, S13b, S13d, S13e and S13f was investigated by Becker et al. Therefore, we can straightforwardly use the algorithmic relations found by the authors when using those boundary conditions: [3]

$\mu_i = \sqrt{\frac{\delta_R}{\delta_i}(\mu_R^2 + m_R^2) - m_i^2}$ (S16a)

$b_1 = \frac{\mu_1}{H_o} a_1$ (S16b)

$Z_1(z_1) = \sin(\mu_1 z_1) + \frac{\mu_1}{H_o}\cos(\mu_1 z_1)$ (S16c)

$Z_1'(z_1) = \mu_1\left(\cos(\mu_1 z_1) - \frac{\mu_1}{H_o}\sin(\mu_1 z_1)\right)$ (S16d)

$Z_i(z_i) = \frac{K_{i-1}}{K_i}\frac{Z_{i-1}'(1)}{\mu_i}\sin(\mu_i z_i) + Z_{i-1}(1)\cos(\mu_i z_i)$ (S16e)

$Z_i'(z_i) = \frac{K_{i-1}}{K_i} Z_{i-1}'(1)\cos(\mu_i z_i) - \mu_i\, Z_{i-1}(1)\sin(\mu_i z_i)$ (S16f)

In turn, equation S10c results in:

$Z_N(1) = 0$ (S16g)

where μ_R and m_R indicates the eigenvalue and the dimensionless perfusion parameter of the reference layer - defined as the one having the minimum value of $\delta_i m_i^2$ (in the present case, the epidermis).[3]

S3. 3 – The stretched exponential function as an approximate solution of the bioheat equation

The values of μ_R can be obtained through equation S16g. At this point it is important to note that μ_R depends on the thermal properties of all layers. As expected, this equation has an infinite number of distinct real roots due to the trigonometric nature of the Z_i functions. Therefore, the solution can be written as:

$$\theta_i(z_i,\tau) = \Psi_i(z_i) - \sum_{n=1}^{\infty} C_n Z_{i,n}(z_i) \times \exp\left(-\delta_R\left(\mu_{R,n}^2 + m_R^2\right)\tau\right)$$

(S17)

Using the definition of θ_i, the temperature in the i-th layer can then be expressed as:

$$T_i(z_i,\tau) = T_a + (T_o - T_{core})\left[\Psi_i(z_i) - \sum_{n=1}^{\infty} C_n Z_{i,n}(z_i) \times \exp\left(-\delta_R\left(\mu_{R,n}^2 + m_R^2\right)\tau\right)\right]$$

(S18)

From which the following relation is obtained:

$$\frac{T_i(z_i,\tau) - T_i(z_i,\infty)}{T_i(z_i,0) - T_i(z_i,\infty)} = \frac{\sum_{n=1}^{\infty} C_n Z_{i,n}(z_i) \times \exp\left(-\delta_R\left(\mu_{R,n}^2 + m_R^2\right)\tau\right)}{\sum_{n=1}^{\infty} C_n Z_{i,n}(z_i)}$$

(S19)

If one realizes that the step, Δn, of the sums in Eq. S16 is equal to 1, then it is easy to see that:

$$\frac{T_i(z_i,\tau) - T_i(z_i,\infty)}{T_i(z_i,0) - T_i(z_i,\infty)} = \frac{\sum_{n=1}^{\infty} C_n Z_{i,n}(z_i) \times \exp\left(-\delta_R\left(\mu_{R,n}^2 + m_R^2\right)\tau\right)\Delta\text{n}}{\sum_{n=1}^{\infty} C_n Z_{i,n}(z_i)\,\Delta\text{n}} \approx$$

$$\frac{\int_1^{\infty} C_n Z_{i,n}(z_i) \times \exp\left(-\delta_R\left(\mu_{R,n}^2 + m_R^2\right)\tau\right) dn}{\int_1^{\infty} C_n Z_{i,n}(z_i)\, dn}$$

(S20)

What equation S20 tells us is that the time-dependent part of the relaxation profile is an average of a multitude of decays characterized by single exponentials. Such a result, however, can be simplified by the relations found by Johnston.[3] They state that a continuous sum of exponential decays can be substituted by the so-called stretched exponential. Therefore,

$$\frac{T_i(z_i,\tau) - T_i(z_i,\infty)}{T_i(z_i,0) - T_i(z_i,\infty)} = e^{-\left(\frac{t}{\tau_r}\right)^{\beta}}$$

(S21)

while τ_r depends on the thermal properties of all the different layers, β is a parameter that defines the probability distribution of the eigenvalues found in S16.

Section S3 - Thermal dependence of physical properties of murine tissues

To obtain this calibration, numerical simulations of the heat diffusion in a computable phantom of a normal male mouse (featuring 43 different tissues, **Figure 5a**) were performed under the consideration of the thermal dependence of physical properties of the liver as previously reported in the literature.[4] The liver was considered to be basically composed of two regions, one corresponding to the illuminated region and the other comprising the non-illuminated one. The illuminated region was modelled in the middle region of the liver as circular spot whose volume corresponded to 10% of the total volume of the organ. The physical and thermal properties of the surrounding tissues were the ones included in the IT'IS Foundation database.[5] The physical properties of the liver, on the other hand were modified according to Manago et al.[6]

Under such conditions, the content of water in the liver was expressed as a function of temperature according to:

$$R_W(T) = -0.298\ \mathrm{T} + 86.0$$

where $R_W(T)$ is the water content ratio, and T the temperature. The heat capacity (c) and thermal conductivity (κ) values, on the other hand, within the same temperature range were worked out from:

$$c(R_W) = 3231\ \times\ 0.2\exp(-0.0269\ \times\ R_W) + 946$$

$$\kappa(R_W) = 0.61 \times\ 0.26\ \exp(-0.0311\ \times\ R_W)$$

The average $R_W(T)$ found in simulations was always around 0.732, with less than a ±10% variation in most of the *in vivo* experiments.

In the liver, blood perfusion was in principle approximated using a piecewise-linear function to account for changes happening in different temperature ranges as thermal relaxation proceeds:

The thermal simulations were performed for 300 seconds. After some preliminary calculations, it was seen that the observed changes in blood perfusion with temperature were always under 1% in the organ of interest. Consequently, a constant value (indicated in **Table 1** of the main text) was adopted.

Section S4 - Algorithm for relaxation time imaging

Relaxation time images (FLIM) are produced by acquiring a series of fluorescence intensity images at a range of time delays after the heating pulse (**Figure S1(a)** and **(b)** and, for each pixel in the field of view, fitting the decay profile to an assumed model. According to the literature, the most suitable model is the one defined by the stretched exponential function (StrEF), also known as the Kohlrausch-Williams-Watts function:

$$I(t) = I_0 \exp\left(-\left(\frac{t}{\tau_k}\right)^{\beta}\right) \qquad \text{(S22)}$$

where I_0 is the initial intensity at $t = 0$, τ_k the characteristic time constant and □ the heterogeneity parameter ($0 < \beta < 1$). The smaller the value of β is, the broader is the distribution of fluorescence lifetimes within a sample, *i.e.* the higher its heterogeneity. In the special case of $\beta = 1$ (minimal heterogeneity) StrEF becomes a single exponential. As one can see, the StrEF is much more appropriate to describe the decay in heterogeneous tissue samples showing continuous lifetime distributions than multi-exponential models with an arbitrary number of discrete relaxation times (which could just as well vary from pixel to pixel). Hence, the computational cost is significantly reduced.

However, to correctly describe the decay, it is necessary to interpret the lifetimes in a statistical manner (notice that τ_k in **Equation S1** is purposefully defined as the characteristic time constant and not the characteristic lifetime). The possibility that we explored was the so called 95-percentile lifetime, *i.e.*, the mean lifetime $<\tau>$ of the distribution as obtained from the integration of Equation S1:

$$< \tau > = \frac{1}{\beta}\, \tau_k \Gamma\left(\frac{1}{\beta}\right) \qquad \text{(S2)}$$

where Γ is the gamma function.

Once the values of $<\tau>$ and I_0 were obtained, two different images were created: the first having $<\tau>$ determining the value of pixels and the second having I_0. The first image received a pseudo coloring treatment (the color scale was suitably chosen to point out the contrast between lifetimes) while the second remained a black and white picture. Having those two images, we multiplied them to produce the final fluorescence lifetime image (**Figure S1(d)**). By multiplying those images, it was ensured that the brightest points on the FLIM were the ones having higher emission.

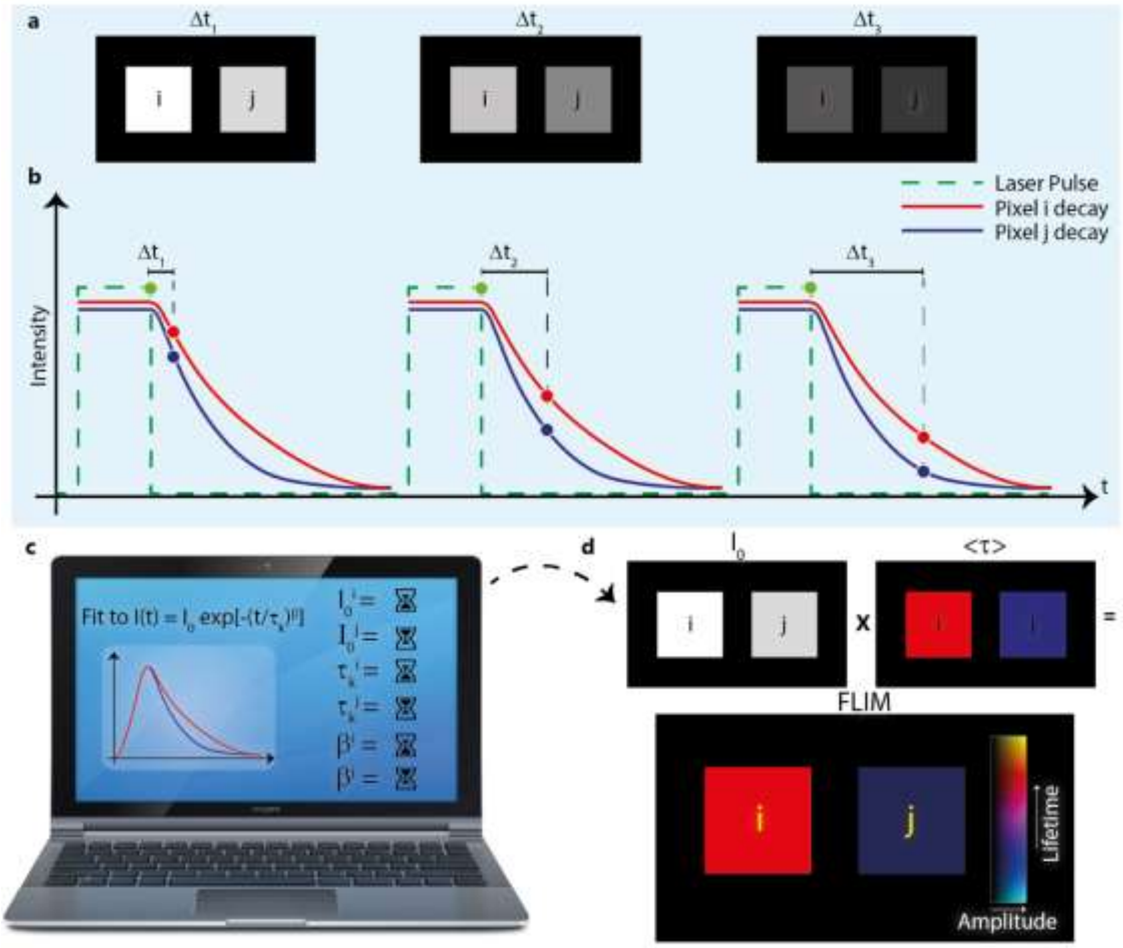

Figure S2. **(a)** Series of fluorescence intensity images. **(b)** Acquisition process. For each fluorescence image obtained at Δt_x, a new point of the decay profile of every pixel is added to the analysis. **(c)** Schematic representation of the computational fit process to the stretched exponential function. **(d)** Multiplication between the amplitude image (black and white) and relaxation time map (colorful) in order to produce the final fluorescence thermal relaxation image.

Section S5 - Postmortem optical images of livers

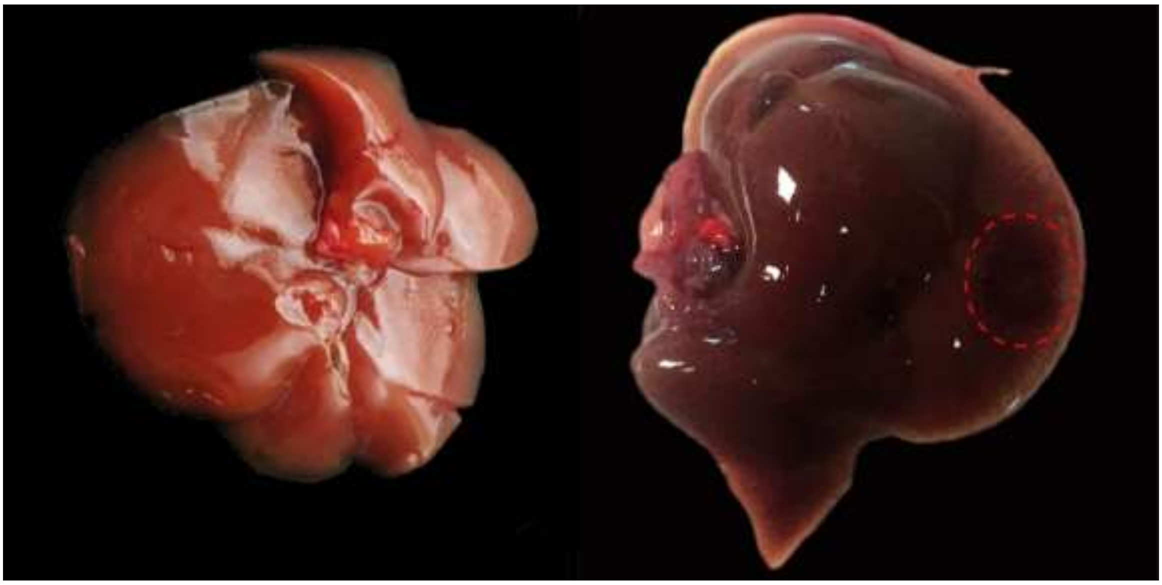

Figure S3. Postmortem optical images of a non-irradiated liver (left) and an irradiated one (right). A small portion of the latter (indicated in the red circle) seems to have been affected by the presence of the laser.

Section S6 – Control of excitation power during the measurement of thermal transients

That is indeed a pertinent question and we apologize if the main text was not sufficiently clear on this matter. The thermal transient experiments consisted of two parts: first, a heating cycle was induced by the 808 nm laser with a high power density (ranging from 100 mW/cm2 up to 900 mW/cm2). This laser is responsible of the liver heating. In a second step, the thermal relaxation of the liver should be recorded by monitoring the intensity generated by the superdots. For doing so, the superdots need to be optically excited and this excitation could, at the same time, induces some extra heating. As perspicaciously noted by the referee, the heating cycle cannot be completely extinguished without switching off the excitation laser. If we did it, no signal would be observed (and therefore, no thermal transient would be recorded). To solve this issue, one must balance two things: the level of signal that is recorded and the undesired heating that is produced. This balance can be achieved by looking for an optimal excitation power density that can be applied in the measurement of the thermal transient. This power density must be high enough to record the intensity variation during relaxation with an acceptable signal-to-noise ratio but low enough to have a negligible induced heating. According to ex-vivo experiments, in which layers of skin and muscle were put on top of a liver (in which the NPs had been accumulating for 22 minutes) and their temperatures were measured with an infrared thermographic camera, the value of power density that satisfied both requirements was found to be 50 mW/cm2. Though the measurement still contains an interference by the low laser-induced heating, its effects are certainly minimized and agree quite well with what is expected from the simulations (Figure 5 of the main text).

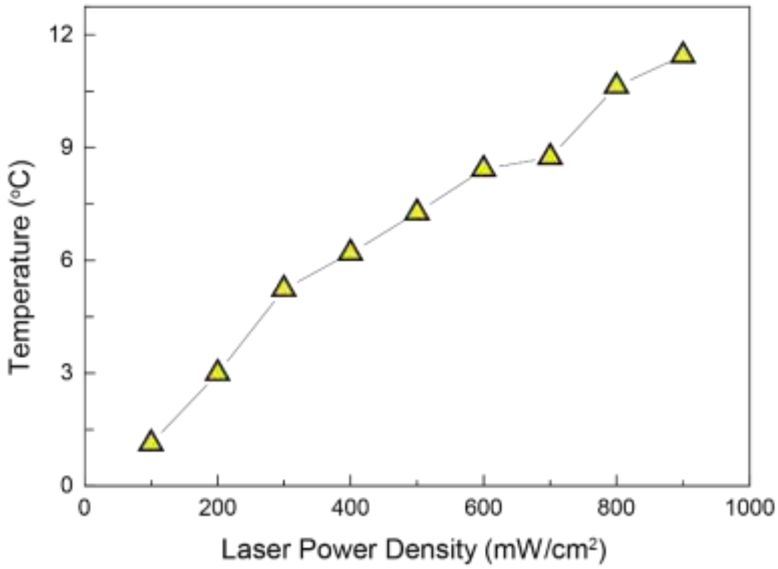

Figure S4. Power density dependence of the temperature variation taking place inside the liver as measured laterally with an infrared thermographic camera.

Section S8 – Additional TEM images of Ag_2S superdots

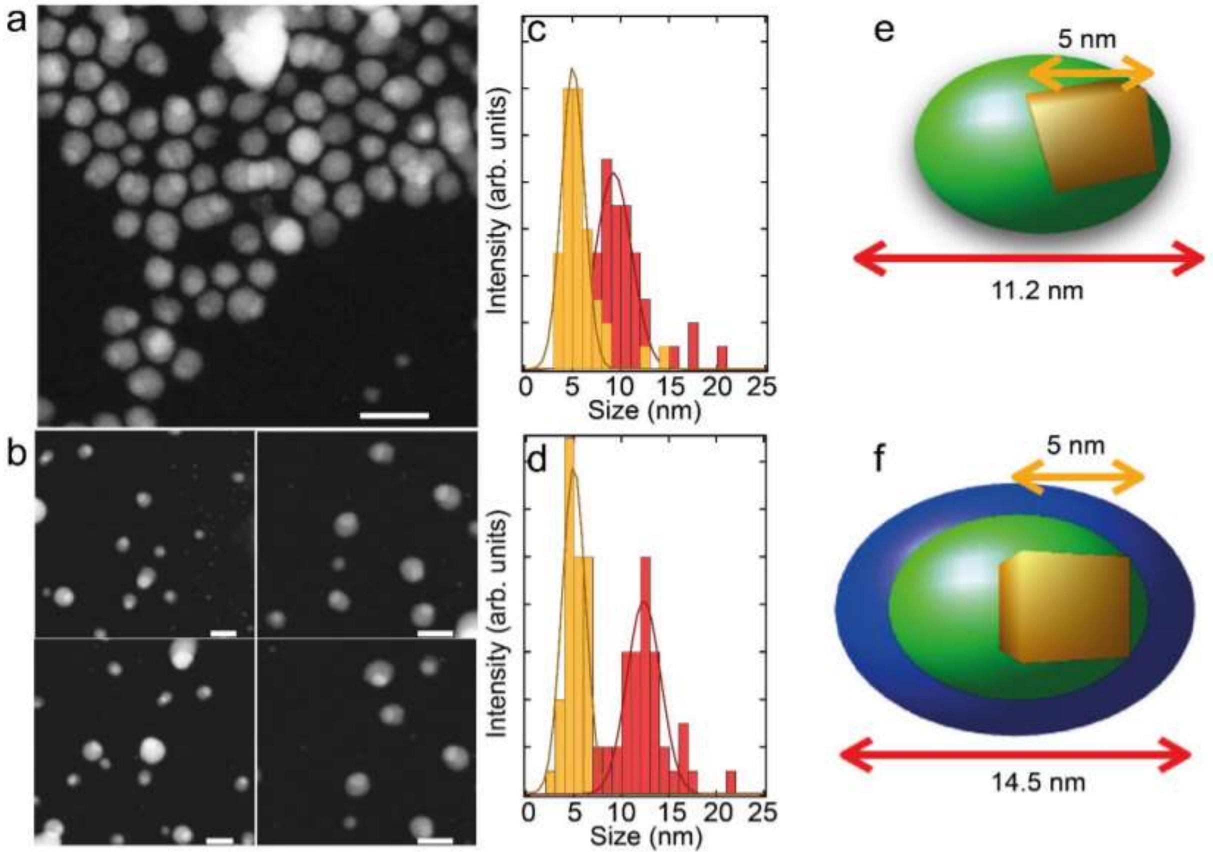

Figure S5. Morphology of Ag2S dots before and after ultrafast laser irradiation. HAADF-STEM image of Ag2S dots (a) and superdots (b). Scale bars are 20 nm. Size distributions of Ag2S dots (c) and superdots (d). Orange and red bars correspond to the size distribution of the Ag core and of the NP, respectively. e Schematic representation of the structure of Ag2S dots. The average sizes of the NP and the Ag core are indicated. f Schematic representation of the structure of Ag2S superdots, including the sizes.

References

[1] D. P. Devor, L. G. DeShazer, R. C. Pastor, *IEEE J. Quantum Electron.* **1989**, *25*, 1863.

[2] T. Y. Fan, *IEEE J. Quantum Electron.* **1993**, *29*, 1457.

[3] D. C. Johnston, *Phys. Rev. B* **2006**, *74*, 184430.

[4] IT'IS Foundation, **2016**, DOI 10.13099/VIP91205-01-0.

[5] IT'IS Foundation, **2018**, DOI 10.13099/VIP21000-04-0.

[6] R. Manago, K. Saito, *IEICE Electron. Express* **2019**, *16*, 20190131.
